# Prevalence and mortality of patients with multiple sclerosis in France in 2012: a study based on French health insurance data

**DOI:** 10.1007/s00415-017-8513-0

**Published:** 2017-05-17

**Authors:** Stéphanie Foulon, Géric Maura, Marie Dalichampt, François Alla, Marc Debouverie, Thibault Moreau, Alain Weill

**Affiliations:** 1 Department of Studies in Public Health, French National Health Insurance, Paris, 75986 Cedex 20 France; 2 General Direction, French National Health Insurance, Paris, France; 3 Department of Neurology, Nancy University Hospital and Inserm CIC 1433, Nancy, France; 4 Department of Neurology, Dijon University Hospital, Dijon, France

**Keywords:** Multiple sclerosis, Epidemiology, Prevalence, Mortality

## Abstract

Data on the prevalence of multiple sclerosis (MS) in France are scarce. National and regional updated estimates are needed to better plan health policies. In this nationwide study, we provided estimates of the prevalence of MS in France in 2012 and mortality rate in 2013. MS cases were identified in the French national health insurance database (SNIIRAM-PMSI) using reimbursement data for disease-modifying treatment, long-term disease status for MS, disability pension for MS, and hospitalisation for MS (MS ICD-10 code: G35). We identified 99,123 MS cases, corresponding to an overall crude prevalence rate of 151.2 per 100,000 inhabitants [95% confidence interval (CI) 150.3–152.2]: 210.0 per 100,000 in women (95% CI 208.4–211.5) and 88.7 per 100,000 in men (95% CI 87.6–89.7). The overall prevalence rate was 155.6 per 100,000 inhabitants (95% CI 154.7–156.6) after standardization on the 2013-European population. We observed a prevalence gradient with a higher prevalence (190–200 per 100,000) in North-Eastern France and a lower prevalence in Southern and Western France (126–140). The crude mortality rate in 2013 was 13.7 per 1,000 MS cases (11.4 in women and 20.3 in men). The standardized mortality ratio was 2.56 (95% CI 2.41–2.72). Our results revise upwards the estimation of MS prevalence in France and confirm the excess mortality of MS patients compared to the general population.

**Electronic supplementary material:**

The online version of this article (doi:10.1007/s00415-017-8513-0) contains supplementary material, which is available to authorized users.

## Introduction

Multiple sclerosis (MS) is a debilitating neurological disease responsible for progressive accumulation of cognitive and neurological impairment, and the corresponding increasing health care and costs. It is essential to have up-to-date estimates of MS prevalence to assess the impact of this chronic condition on the health care system and to allocate national and regional resources for the management of MS patients. MS has a heterogeneous prevalence in Europe [1, 2]. Only a few studies have investigated the prevalence of MS in France, providing estimates of national prevalence [3, 4], or local prevalence [5, 6]. In view of the marked geographical disparities of the prevalence of MS in France, the French national prevalence cannot be extrapolated from local estimates and studies on comprehensive nationwide data are needed. The most recent estimate of MS prevalence in France was based on 2004 data concerning Long-Term Diseases (LTD) recorded in the French Health Insurance database. In France, LTD registration allows patients with certain diseases and particularly costly therapies, such as MS, to be fully reimbursed (100%) for all MS outpatient and hospital healthcare.

Since 2010, French healthcare databases have been increasingly used for epidemiological purposes [7]. In addition to demographics and LTD status for MS, further data are now available in these databases, namely, reimbursements for disease-modifying treatments, disability pensions for MS, and hospitalisation for MS. This has improved the identification of MS patients in healthcare databases and estimates of MS prevalence.

Mortality is significantly higher in MS patients compared to the general population, but the excess mortality varies between 1.3- and 3-fold depending on the selection of the MS population (clinical centre, registry, or insurance based), the duration of follow-up, the study period, and the geographical area [8–11]. To date, no nationwide study on the mortality of MS patients has been performed in France.

The present study was designed to estimate the prevalence of MS in France on 31 December 2012 and the mortality rate of these patients in 2013, using the French National Health Insurance database.

## Materials and methods

### Design and data sources

This was a cross-sectional study using data from the French National Health Insurance database (SNIIRAM) linked with the National hospital discharge database (PMSI) [12]. The SNIIRAM database contains individualized, anonymous and comprehensive data on all reimbursements of patients’ health expenditure. Data are derived from the various French Health Insurance schemes, including the General scheme and the various special insurance schemes. Altogether, these schemes cover nearly the entire population residing in France (65.5 million inhabitants in 2012). Besides data on health expenditure reimbursements, demographic data are available, including the year of birth, gender, area of residence, geographical deprivation [13], and date of death. The SNIIRAM also contains information on patient eligibility for 100% health insurance coverage for LTD encoded in the International Classification of Diseases, 10th Revision (ICD-10). Similar diagnostic coding is used for the payment of disability pensions for patients of the general scheme. The SNIIRAM database is linked by a unique patient identifier to the PMSI database which provides medical information about all private and public hospital stays in France, including hospitalisation dates and diagnoses coded according to ICD-10.

### Algorithm to identify MS cases

We defined four selection criteria to identify a prevalent MS case: reimbursement of a disease-modifying treatment, LTD for MS, disability pensions for MS, and hospitalisation for MS. Since MS is a chronic disease with low or no relapse activity for several years, our selection period was not limited to 2012. To maximize the chances of identifying MS cases, we extended the identification period back in time according to the maximum data storage period available for this study in the databases, i.e., 2011–2012 for SNIIRAM and 2008–2012 for the PMSI.

The selection criteria were: (1°) at least one reimbursement for a disease-modifying treatment (interferon beta, glatiramer acetate, fingolimod, or natalizumab); (2°) LTD for MS with at least one reimbursement related or not related to MS; (3°) at least one disability pension payment for MS (general scheme patients only); and (4°) at least one hospitalisation with a discharge diagnosis of MS (principal, related or associated diagnoses). The ICD-10 code used for these three latter criteria was “G35”. An individual was identified as a prevalent MS case if (1°) he/she met at least one of the four selection criteria and (2°) was alive on 31 December 2012 and (3°) had at least one reimbursement in 2012. This latter criterion was added to ensure that MS patients had received reimbursed care on the national territory in 2012.

### Statistical analysis

#### Prevalence

The national prevalence of multiple sclerosis was defined as the number of MS cases identified on 31 December 2012 divided by the number of people residing in France (including overseas departments) on 1st January 2013 (http://www.insee.fr). The national prevalence rate was then standardized by gender and by 5-year age category to the 1976 European population, the 2013 European population, and the world population, for comparison purposes.

Geographical variations of MS prevalence were studied using standardized prevalence rates by department and grouped by administrative regions. The reference population for standardization was the general population residing in France on January 1, 2013.

#### Mortality rate

Patients of the MS population on 31 December 2012 who died in 2013 were identified. Age at death and crude mortality rate in 2013 were calculated. A standardized mortality ratio (SMR) was calculated to compare the mortality rate of MS cases with that of the French general population in 2012. This mortality analysis was limited to general scheme beneficiaries (75% of the population living in France) as the date of death is comprehensively reported by this scheme in the SNIIRAM database, in contrast with the other health insurance schemes.

## Results

### Description of the population

On 31 December 2012, 99,123 patients with MS in France were identified from the SNIIRAM/PMSI databases and 68.4% of these patients were identified by at least two selection criteria. LTD status for MS allowed the identification of 83.3% of MS cases, followed by hospitalisation (69.6%), reimbursement of a disease-modifying treatment (40.1%) and payment of a disability pension for MS (16.3%). 13.5% of the identified MS cases were captured only through hospitalisation records. The sex ratio (female/male) was 2.5. The mean age of the population identified on 31 December 2012 was 50.3 ± 14.2 years and one half of MS cases were between the ages of 40.0 and 60.0 years. 78% of MS cases in metropolitan France lived in urban areas. The distribution of MS cases according to geographical deprivation index was similar to that of the general population.

### Prevalence

On 31 December 2012, the national crude prevalence of multiple sclerosis in France was 151.2 per 100,000 inhabitants [95% confidence interval (CI) 150.3–152.2] 210.0 per 100,000 in women (95% CI 208.4–211.5) and 88.7 per 100,000 in men (95% CI 87.6–89.7). The national prevalence rate based on the information provided by LTD status only was 125.9 per 100,000 inhabitants. The crude prevalence rates by gender and age-group are shown in Table 1. The peak prevalence of MS occurred between the ages of 50 and 54 years in women and 55 and 59 years in men. Prevalence rates after standardization on the European and World populations are presented in Table 2 to allow international comparisons.

**Table 1:** Age-specific and gender-specific crude prevalence rates of multiple sclerosis (MS) in France, in 2012

Age	Number of MS cases	Number of people residing in France	Overall		Women		Men	
			MS crude prevalence	95% confidence interval	MS crude prevalence	95% confidence interval	MS crude prevalence	95% confidence interval
Total France	99,123	65,542,916	151.2	150.3–152.2	210.0	208.4–211.5	88.7	87.6–89.7
Under 15	136	12,164,564	1.1	0.9–1.3	1.3	1.0–1.6	0.9	0.7–1.2
15–19	490	3,957,475	12.4	11.3–13.5	17.7	15.8–19.6	7.3	6.1–8.5
20–24	1821	3,963,556	45.9	43.8–48.1	64.2	60.7–67.8	27.9	25.6–30.3
25–29	4481	3,925,169	114.2	110.8–117.5	166.2	160.5–171.8	60.7	57.2–64.2
30–34	7416	4,105,931	180.6	176.5–184.7	256.7	249.9–263.6	102.1	97.7–106.5
35–39	9113	4,164,014	218.9	214.4–223.3	313.5	306.0–321.1	122.8	118.0–127.6
40–44	11,542	4,559,827	253.1	248.5–257.7	360.8	353.0–368.5	143.7	138.7–148.6
45–49	13,213	4,542,473	290.9	285.9–295.8	411.1	402.9–419.4	167.1	161.7–172.4
50–54	12,998	4,364,041	297.8	292.7–303.0	425.9	417.3–434.4	164.3	158.9–169.7
55–59	11,672	4,173,094	279.7	274.6–284.8	384.5	376.2–392.8	168.1	162.4–173.7
60–64	10,391	4,108,440	252.9	248.1–257.8	337.6	329.8–345.3	161.7	156.1–167.3
65–69	6666	3,221,653	206.9	202.0–211.9	271.7	263.9–279.6	135.5	129.7–141.3
70–74	3827	2,368,290	161.6	156.5–166.7	212.5	204.6–220.5	101.3	95.3–107.3
75–79	2712	2,228,492	121.7	117.1–126.3	154.6	147.8–161.4	77.4	71.9–83.0
80–84	1641	1,864,716	88.0	83.7–92.3	109.0	103.0–115.1	54.4	49.0–59.8
85 plus	860	1,831,181	47.0	43.8–50.1	54.0	49.9–58.0	30.9	26.3–35.5

**Table 2:** Number of multiple sclerosis (MS) cases and prevalence rates in France, in 2012

	Overall	Women	Men
Number of MS cases	99,123	70,963	28,148
2012 population	65,542,916	33,795,692	31,747,224
Crude prevalence rates per 100,000 population	151.2 (150.3–152.2)	210.0 (208.4–211.5)	88.7 (87.6–89.7)
Standardized prevalence rates per 100,000 world population	117.4 (116.6–118.1)	165.9 (164.6–167.1)	68.9 (68.0–69.7)
Standardized prevalence rates per 100,000 1976-European population	144.1 (143.2–145.0)	203.3 (201.8–204.8)	85.0 (83.9–86.0)
Standardized prevalence rates per 100,000 2013-European population	155.6 (154.7–156.6)	218.5 (216.9–220.2)	92.7 (91.6–93.8)

**Fig. 1:**
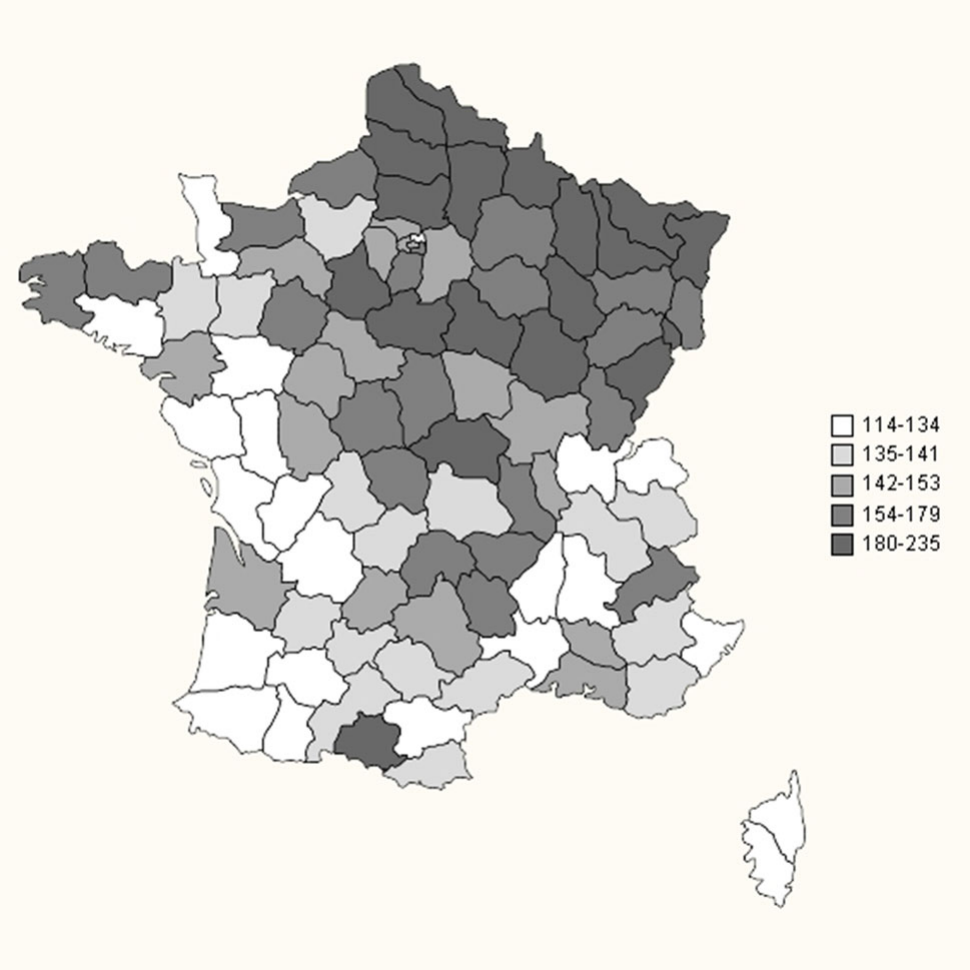
Map of standardized prevalence rates of multiple sclerosis in France in each department (except for overseas departments, whose results are presented in Table 3)

The standardized prevalence in each region of France is shown in Table 3 and represented graphically in Fig. 1 for each department. Higher standardized prevalence rates were observed in North-Eastern regions of France (e.g., Lorraine, Picardie, or Alsace, with close to 200 MS cases per 100,000 inhabitants) than in South-Western regions (Languedoc-Roussillon, Corse, and Poitou–Charentes with approximately 130 MS cases per 100,000 inhabitants). The standardized prevalence rates in French overseas departments were considerably lower, ranging from 22.9 in Reunion Island (Indian Ocean) to 49.4 MS cases per 100,000 inhabitants in Martinique (Caribbean).

**Table 3:** Standardized prevalence rates of multiple sclerosis (MS) on 31 December 2012 in each region of France

Administrative region of France	Number of MS cases	Number of people living in the region	Overall		Women		Men	
			MS standardized prevalence	95% confidence interval	MS standardized prevalence	95% confidence interval	MS standardized prevalence	95% confidence interval
Total France^a^	99,123	65,542,916	151.2	150.3–152.2	210.0	208.4–211.5	88.7	87.6–89.7
Lorraine	4785	2,350,657	200.2	194.6–205.9	277.9	268.6–287.3	117.5	111.3–123.7
Picardie	3701	1,924,737	193.8	187.5–200.0	272.7	262.4–283.1	109.7	102.9–116.4
Alsace	3636	1,861,020	192.6	186.3–198.8	269.5	259.2–279.9	110.7	103.9–117.4
Nord-Pas-de-Calais	7531	4,052,156	190.6	186.3–194.9	255.0	248.1–261.9	122.0	117.1–127.0
Champagne-Ardenne	2544	1,333,497	190.3	182.9–197.7	267.6	255.3–279.8	108.0	100.0–115.9
Franche-Comté	2180	1,177,906	185.7	177.9–193.5	270.6	257.4–283.7	95.4	87.4–103.3
Bourgogne	2855	1,643,931	170.1	163.8–176.4	247.2	236.7–257.7	88.1	81.6–94.5
Centre	4385	2,572,931	169.3	164.3–174.3	239.0	230.7–247.3	95.1	89.7–100.5
Auvergne	2170	1,355,630	155.0	148.4–161.6	224.9	213.8–235.9	80.6	73.9–87.3
Haute-Normandie	2779	1,848,102	150.8	145.2–156.4	205.4	196.3–214.5	92.7	86.4–99.1
Ile-de France	17,413	11,978,363	149.2	147.0–151.4	202.1	198.4–205.7	92.9	90.4–95.5
Basse-Normandie	2213	1,479,242	148.0	141.8–154.2	205.6	195.4–215.8	86.7	79.9–93.4
Bretagne	4786	3,259,659	146.6	142.4–150.8	206.6	199.7–213.5	82.7	78.2–87.1
Limousin	1118	741,047	144.5	136.0–153.1	210.3	195.9–224.7	74.5	65.8–83.3
Midi-Pyrénées	4223	2,946,507	141.2	136.9–145.5	199.2	192.1–206.2	79.5	74.9–84.0
Provence-Alpes-Côte d’Azur	7128	4,937,445	141.2	137.9–144.4	193.9	188.5–199.2	85.1	81.4–88.8
Rhône-Alpes	8862	6,393,470	140.4	137.4–143.3	192.6	187.9–197.4	84.7	81.4–87.9
Pays de la Loire	5019	3,658,351	139.4	135.6–143.3	200.3	193.9–206.8	74.6	70.5–78.6
Aquitaine	4669	3,303,392	137.1	133.2–141.0	193.4	186.9–199.9	77.2	72.9–81.4
Languedoc-Roussillon	3689	2,727,286	133.1	128.7–137.4	186.6	179.5–193.7	76.0	71.3–80.7
Poitou–Charentes	2401	1,792,159	130.1	124.9–135.3	186.6	177.8–195.3	70.0	64.5–75.5
Corse	422	322,120	125.6	113.6–137.6	176.2	156.3–196.1	71.8	58.9–84.8
Martinique	207	386,486	49.4	42.5–56.2	69.8	58.8–80.8	27.6	19.7–35.4
Guadeloupe	159	405,739	37.7	31.8–43.7	51.4	42.0–60.9	23.1	16.0–30.3
Guyane	39	250,109	24.6	15.1–34.0	35.0	19.2–50.7	13.6	3.5–23.6
Réunion	177	840,974	22.9	19.3–26.4	26.7	21.3–32.0	18.8	14.2–23.5

^a^Crude prevalence of MS

### Mortality rate

**Fig. 2:**
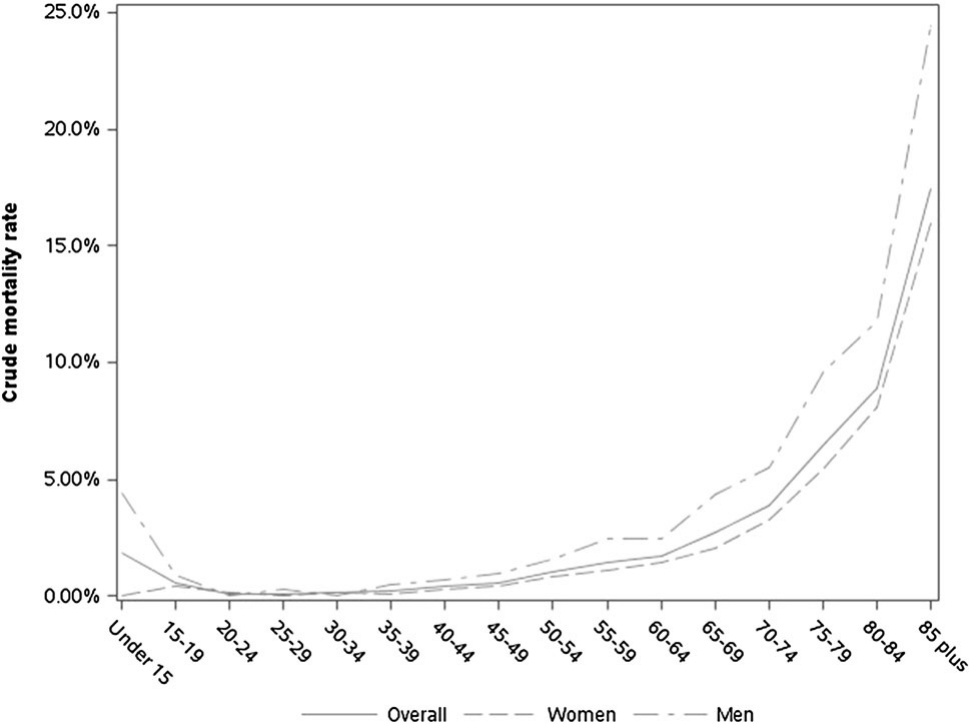
Age-specific and gender-specific crude mortality rates in 2013, among multiple sclerosis cases

Among the 78,805 general scheme beneficiaries identified as MS cases, 1080 died in 2013. The mean age at death was 66.6 ± 13.9 years (68.2 ± 13.9 in women and 64.1 ± 13.5 in men). The crude mortality rate in 2013 for people identified as having MS on 31 December 2012 was 13.7 per 1000 MS cases (11.4 in women and 20.3 in men). Age-specific and gender-specific crude mortality rates are presented in Fig. 2. The SMR, as compared with the general population, was 2.56 (95% CI 2.41–2.72). The SMR was 2.55 (95% CI 2.35–2.75) in women and 2.58 (95% CI 2.34–2.83) in men.

## Discussion

### Principal findings of the study

The French MS prevalence was estimated at 151.2 per 100,000 inhabitants (prevalence rate of 155.6/100,000 standardized to the European population) in 2012 based on data from the French healthcare databases including ICD-10 G35 diagnoses from LTD, hospitalisations, disability pensions, and reimbursements of MS disease-modifying treatment. Patients with MS had a 2.5-fold increased risk of all-cause mortality compared with the general population.

### Comparison with French and European estimates of MS prevalence in the literature

Table 4 summarizes the methodologies and results of the previous studies carried out in France to estimate the MS prevalence at a national or local level, presenting their respective strengths and limits. The estimations (per 100,000 inhabitants) based on the capture-recapture method in Haute-Garonne (138–149) and in Lorraine (188.2) are close to our estimations (141.8 and 200.2, respectively).

**Table 4:** Previous studies on MS prevalence in France

Author	Year of the study	Data sources and methodology	MS prevalence (per 100,000 inhabitants) with its 95% confidence interval, when available	Strengths and limits
Vukusic [4]	2003	Identification of MS patients using the LTD diagnosis in the French agricultural workers health insurance system database. Prevalence standardized using the age structure of the French population	Age-standardized prevalence: 65.0 (62.5–67.5)	Strengths: nationwide estimation Limits: lack of representativeness. Only 7% of the French population is covered by the agricultural workers health insurance system. LTD status for MS may not be sufficiently sensitive to identify all MS cases
Fromont [3]	2004	Identification of MS patients using the LTD diagnosis in the General Scheme health insurance system database. Prevalence standardized using the age structure of the French population	Age-standardized prevalence: 94.7 (94.3–95.1) Higher rates in North-Eastern versus South-Western regions	Strengths: nationwide estimation, population more representative of the French population than in Vukusic paper Limits: LTD status for MS may not be sufficiently sensitive to identify all MS cases
Sagnes-Raffy [6]	2005	Identification of MS patients by matching several data sources by a capture-recapture method in Haute Garonne, a department in the South West of France. The data were hospital data, LTD status and reimbursement of specific treatments of MS using local health insurance data, and data from a MS health network	Modelled prevalence using the capture-recapture method: 138–149	Strengths: multisource and independent data collection Limits: local estimation
El Adssi [5]	2008	Identification of MS patients by matching several data sources by a capture-recapture method in Lorraine, a region in the North East of France. The data were hospital data, LTD status and reimbursement of specific treatments of MS using local health insurance data, and data from the Lorraine registry of MS	Crude prevalence: 170.9 (165.7–176.3) Modelled prevalence using the capture-recapture method: 188.2 (182.7–193.8)	Strengths: multisource and independent data collection Limits: local estimation

Despite many studies on the prevalence of multiple sclerosis in European countries, published data are not sufficient to allow reliable comparison between European countries. This is essentially due to a lack of standardized estimates as published rates are rarely age-and-sex-adjusted to the European population. Furthermore, prevalence estimates are often provided for regions or cities in a given country and some European regions are underrepresented. The methodological quality of epidemiological studies, therefore, is highly variable but has improved in the more recent studies due to the use of administrative databases [2, 14]. When searching for recently published estimates of MS prevalence in neighbouring countries of France, very few studies were found to provide MS prevalence rates standardized to the European population [15–19]. Although no direct comparison can, therefore, be established, these recent data suggest that our standardized MS prevalence rate of 155.6 per 100,000 for the 2013 European population is in an intermediate position between those reported in studies from northern (203.4 and 175 per 100,000 in UK and Germany) and southern (96.0 and 70.6 per 100,000 in Italy and Spain) neighbouring countries.

### French regional variations in MS prevalence

France is located in the middle of Western Europe. We observed a prevalence gradient with a higher prevalence (190–200 per 100,000) in North-Eastern France and a lower prevalence in the Southern (126 per 100,000 in Corsica-Mediterranean island) and South-Western France (130–140 per 100,000). Age-and-sex-standardized rates determined by an identical method of measurement at the same date from the claims database of a homogeneous national health system support our geographical comparisons according to region. The prevalence gradient observed in our study corresponding to about seven additional cases per 100,000 inhabitants by degree of latitude is in line with that observed by Simpson et al. for Western Europe (8.1 cases per 100,000 inhabitants by degree of latitude) [20]. The standardized prevalence gradient appears to be slightly more marked than in 2004 [3], although classification of regions according to MS prevalence remains globally similar. The present study is unable to determine the respective roles of possible ethnic or environmental factors that might explain this gradient.

### Mortality in MS patients

We found an excess mortality in MS patients compared to the general population with an SMR of 2.56. This result is consistent with the international literature [8] in which SMR ranges between 2.47 and 2.89, but differs from the result (SMR 1.48) published by Leray et al. [11]. This difference can be explained by the mean follow-up period which was only 15 ± 10 in this study, compared with the longer follow-up period reported in other international studies. In our study, the MS population was followed for only 1 year, but all of the prevalent MS populations were followed, therefore, including young, recent diagnosed patients as well as patients with a longstanding diagnosis.

While crude mortality rates were higher in men than in women, SMRs were similar in both sexes, suggesting that sex-and-age-related differences in MS mortality simply reflect sex-and-age-related differences in the general population, unrelated to the disease.

### Strengths and limitations

To our knowledge, this is the first study to provide such an accurate estimate of French MS prevalence and one of the largest studies of national MS prevalence. One of the main strengths of this study is to provide regional estimates of MS prevalence using identical method of measurement. It facilitates regional comparisons and can be helpful for health decision makers to better plan health policies according to MS prevalence.

The prevalent MS population was identified using health insurance reimbursement data from two databases (SNIIRAM and PMSI) with completely independent data collection. Controls insuring the quality of data exist for each of the selection criteria. Information on reimbursement of drugs is collected by entering the specific bar code and transmitted thanks to the patient’s personal smart card. The high cost of treatments for MS excludes the hypothesis of non-reimbursed health care. LTD status for MS is requested by the patient’s GP and validated by the health insurance medical adviser who codes the diagnosis according to ICD-10. A similar process applies to disability pension. Similarly, hospitalisation data are systematically recorded by hospital physicians after hospitalisation to determine the cost of each hospital stay. Physicians specialised in these codes validate and transmit this information to paying bodies. Controls are performed to ensure the validity of the ICD—10 codes used. However, the SNIIRAM-PMSI databases have been created for payment management purposes. Just like other claims and hospitalisation databases, its main limitation is the lack of case validation. The anonymization of the data makes it difficult to match clinical cohort data with SNIIRAM-PMSI data to perform such case validation. As explained by Moulis et al., in the PMSI, only events accompanying the diagnosis or complicating the disease are encoded. Moreover, LTD attributions are not exhaustive for a given disease. If a patient has already obtained an LTD for a certain disease, he may not systematically request a new LTD for another disease. Yet, in our study, the number of information sources observed for each case identified (an average of almost two) and the closeness of our results and the estimates provided by the regional studies with case validations in Haute-Garonne and Lorraine are convincing elements for the quality of the information. With a 5-year pre-index period to detect hospitalisation diagnoses and a 2-year period to detect other criteria, our MS prevalence estimates are probably underestimated by at least 15% as MS cases characterized by a single relapse or benign MS may have been missed. Missing benign MS cases may slightly impact the mortality estimates. Benign MS cases may be less likely to die prematurely compared to other MS cases. Therefore, this bias could have led to a slight overestimation of the mortality of MS patients in our study.

MS incidence was not estimated in this study as identification of incident cases from claims data is a challenging process requiring a long patient history of diagnosis and treatment. Previous studies have reported that a 4-year history is insufficient to accurately classify MS patients as incident on the basis of claims data [21].

## Conclusion

Based on national health insurance data covering of the French population, the prevalence of MS was 155.6 per 100,000 inhabitants (95% CI 154.7–156.6) after standardization on the 2013-European population. This estimation is higher than that previously described. After standardization on sex and age, MS patients had a 2.5-fold increased risk of all-cause mortality compared with the general population.

This database, comprising almost 100,000 people with MS in a single country, could be used to more clearly understand care pathways and costs of management or be linked with the European Database of Multiple Sclerosis (EDMUS) to combine the respective advantages of these two data sources.

## Electronic supplementary material

Below is the link to the electronic supplementary material.

Supplementary material 1 (DOCX 47 kb)
