# Supplementary material for: Prevalence and mortality of patients with multiple sclerosis in France in 2012: a study based on French health insurance data
Source: J Neurol. 2017 May 17;264(6):1185–92. doi: 10.1007/s00415-017-8513-0 (PMC5486573; doi:10.1007/s00415-017-8513-0)
Supplement: Supplementary file 1 — Supplementary material 1 (DOCX 47 kb) [file 415_2017_8513_MOESM1_ESM.docx]

# Supplemental material

Table S1 Standardised prevalence rates of multiple sclerosis on 31 December 2012 in each department of France

|  |  |  | Overall | | Women | | Men | |
| --- | --- | --- | --- | --- | --- | --- | --- | --- |
| Administrative department of France | Number of MS cases | Number of people living in the department | MS standardised prevalence | 95% confidence interval | MS standardised prevalence | 95% confidence interval | MS standardised prevalence | 95% confidence interval |
| Total France^a^ | 99,123^b^ | 65,542,916 | 151.2 | [150.3-152.2] | 210.0 | [208.4-211.5] | 88.7 | [87.6-89.7] |
| 01-Ain | 757 | 618,632 | 124.1 | [115.2-133.0] | 178.3 | [163.4-193.1] | 66.5 | [57.3-75.6] |
| 02-Aisne | 1,065 | 542,550 | 196.5 | [184.7-208.3] | 275.0 | [255.5-294.5] | 112.9 | [100.1-125.6] |
| 03-Allier | 657 | 342,534 | 184.2 | [170.0-198.5] | 269.3 | [245.2-293.4] | 93.7 | [79.1-108.2] |
| 04-Alpes-de-Haute-Provence | 236 | 162,923 | 137.8 | [119.9-155.7] | 199.5 | [169.4-229.5] | 72.1 | [53.9-90.4] |
| 05-Hautes-Alpes | 220 | 141,417 | 154.3 | [133.7-175.0] | 219.7 | [185.2-254.2] | 84.7 | [63.1-106.4] |
| 06-Alpes-Maritimes | 1,504 | 1,078,874 | 135.0 | [128.1-141.8] | 192.2 | [180.8-203.5] | 74.1 | [66.7-81.5] |
| 07-Ardèche | 416 | 320,924 | 132.6 | [119.7-145.5] | 189.5 | [167.9-211.0] | 72.0 | [58.4-85.6] |
| 08-Ardennes | 582 | 281,866 | 204.1 | [187.5-220.7] | 274.8 | [247.9-301.7] | 128.9 | [110.1-147.7] |
| 09-Ariège | 273 | 153,453 | 187.8 | [165.0-210.5] | 273.1 | [235.0-311.2] | 96.9 | [73.4-120.4] |
| 10-Aube | 549 | 305,485 | 179.6 | [164.6-194.7] | 252.6 | [227.6-277.5] | 102.0 | [85.8-118.2] |
| 11-Aude | 470 | 366,604 | 125.8 | [114.4-137.3] | 183.1 | [163.8-202.4] | 64.9 | [53.0-76.7] |
| 12-Aveyron | 405 | 275,487 | 142.3 | [128.2-156.3] | 200.7 | [177.3-224.2] | 80.0 | [65.1-94.9] |
| 13-Bouches-du-Rhône | 2,902 | 1,982,423 | 145.6 | [140.3-150.9] | 193.2 | [184.7-201.6] | 95.0 | [88.7-101.2] |
| 14-Calvados | 1,089 | 688,887 | 158.1 | [148.7-167.5] | 216.7 | [201.4-232.0] | 95.6 | [85.1-106.1] |
| 15-Cantal | 266 | 146,464 | 173.2 | [152.0-194.4] | 258.8 | [222.5-295.1] | 82.2 | [61.6-102.7] |
| 16-Charente | 462 | 353,140 | 125.7 | [114.1-137.3] | 173.0 | [154.0-191.9] | 75.4 | [62.7-88.2] |
| 17-Charente-Maritime | 886 | 634,191 | 132.4 | [123.6-141.2] | 190.3 | [175.6-205.1] | 70.8 | [61.5-80.0] |
| 18-Cher | 519 | 310,370 | 162.8 | [148.6-176.9] | 244.4 | [220.2-268.7] | 75.8 | [62.3-89.4] |
| 19-Corrèze | 351 | 241,986 | 139.3 | [124.5-154.1] | 210.7 | [185.2-236.2] | 63.3 | [49.2-77.3] |
| 21-Côte-d'Or | 1,034 | 528,403 | 196.1 | [184.1-208.0] | 283.3 | [263.2-303.3] | 103.3 | [90.8-115.7] |
| 22-Côtes-d'Armor | 1,021 | 601,822 | 167.1 | [156.7-177.5] | 230.5 | [213.5-247.6] | 99.5 | [88.2-110.9] |
| 23-Creuse | 228 | 121,579 | 170.4 | [147.8-193.1] | 257.3 | [218.3-296.3] | 78.0 | [56.6-99.5] |
| 24-Dordogne | 579 | 418,806 | 128.8 | [118.1-139.5] | 185.0 | [167.0-202.9] | 69.1 | [57.9-80.2] |
| 25-Doubs | 962 | 532,549 | 184.3 | [172.7-196.0] | 265.3 | [245.8-284.8] | 98.2 | [86.1-110.3] |
| 26-Drôme | 679 | 494,385 | 134.7 | [124.5-144.9] | 182.8 | [166.3-199.2] | 83.5 | [72.0-95.1] |
| 27-Eure | 843 | 595,310 | 141.5 | [132.0-151.1] | 193.5 | [177.9-209.1] | 86.2 | [75.6-96.9] |
| 28-Eure-et-Loir | 832 | 434,711 | 191.3 | [178.3-204.3] | 264.7 | [243.4-286.1] | 113.2 | [99.0-127.4] |
| 29-Finistère | 1,510 | 905,164 | 165.0 | [156.6-173.3] | 236.7 | [222.7-250.7] | 88.6 | [79.9-97.3] |
| 2A-Corse-du-Sud | 183 | 149,013 | 116.8 | [99.8-133.8] | 160.3 | [132.4-188.1] | 70.6 | [51.6-89.5] |
| 2B-Haute-Corse | 239 | 173,107 | 134.0 | [117.0-151.0] | 190.9 | [162.5-219.2] | 73.5 | [55.6-91.3] |
| 30-Gard | 878 | 733,747 | 117.3 | [109.5-125.0] | 165.9 | [153.1-178.8] | 65.4 | [57.1-73.8] |
| 31-Haute-Garonne | 1,790 | 1,289,664 | 141.8 | [135.2-148.4] | 195.6 | [184.8-206.3] | 84.5 | [77.2-91.8] |
| 32-Gers | 261 | 191,082 | 128.6 | [112.7-144.5] | 192.0 | [164.8-219.2] | 61.1 | [45.6-76.6] |
| 33-Gironde | 2,217 | 1,491,170 | 147.6 | [141.4-153.7] | 205.9 | [195.8-216.0] | 85.5 | [78.7-92.2] |
| 34-Hérault | 1,545 | 1,090,052 | 141.1 | [134.1-148.2] | 194.4 | [182.9-205.9] | 84.4 | [76.5-92.3] |
| 35-Ille-et-Vilaine | 1,339 | 1,015,427 | 136.5 | [129.2-143.8] | 190.6 | [178.5-202.7] | 78.9 | [70.9-86.9] |
| 36-Indre | 430 | 229,015 | 179.2 | [162.0-196.4] | 249.8 | [221.5-278.1] | 104.0 | [85.2-122.9] |
| 37-Indre-et-Loire | 902 | 598,872 | 151.3 | [141.4-161.1] | 214.1 | [197.7-230.4] | 84.4 | [73.8-95.0] |
| 38-Isère | 1,648 | 1,232,257 | 136.1 | [129.5-142.7] | 180.3 | [169.7-190.9] | 89.0 | [81.5-96.6] |
| 39-Jura | 447 | 261,174 | 169.4 | [153.6-185.1] | 254.1 | [227.2-281.1] | 79.2 | [63.9-94.4] |
| 40-Landes | 534 | 397,611 | 127.0 | [116.1-137.8] | 182.3 | [164.2-200.5] | 68.0 | [56.7-79.3] |
| 41-Loir-et-Cher | 511 | 334,319 | 150.9 | [137.7-164.1] | 211.1 | [189.4-232.8] | 86.8 | [72.5-101.1] |
| 42-Loire | 1,285 | 753,550 | 171.7 | [162.3-181.1] | 240.8 | [225.3-256.3] | 98.1 | [87.9-108.3] |
| 43-Haute-Loire | 362 | 226,685 | 154.4 | [138.3-170.4] | 218.4 | [191.6-245.2] | 86.2 | [69.3-103.0] |
| 44-Loire-Atlantique | 1,850 | 1,322,404 | 142.2 | [135.8-148.7] | 200.6 | [189.9-211.4] | 80.0 | [73.0-87.0] |
| 45-Loiret | 1,191 | 665,644 | 181.0 | [170.7-191.3] | 253.6 | [236.6-270.5] | 103.7 | [92.6-114.9] |
| 46-Lot | 273 | 175,860 | 142.7 | [125.3-160.1] | 208.3 | [178.9-237.7] | 72.8 | [55.3-90.4] |
| 47-Lot-et-Garonne | 479 | 333,569 | 138.4 | [125.9-151.0] | 188.9 | [168.5-209.3] | 84.7 | [70.6-98.8] |
| 48-Lozère | 139 | 77,085 | 172.5 | [143.5-201.6] | 252.7 | [202.8-302.6] | 87.2 | [59.3-115.2] |
| 49-Maine-et-Loire | 987 | 800,424 | 128.0 | [120.1-136.0] | 184.8 | [171.5-198.2] | 67.6 | [59.3-75.9] |
| 50-Manche | 690 | 500,980 | 135.0 | [124.8-145.1] | 193.9 | [176.8-210.9] | 72.3 | [61.9-82.8] |
| 51-Marne | 1,012 | 566,417 | 180.0 | [168.9-191.1] | 255.2 | [236.8-273.6] | 99.9 | [88.0-111.8] |
| 52-Haute-Marne | 401 | 179,729 | 219.3 | [197.7-240.9] | 322.6 | [285.8-359.3] | 109.3 | [87.9-130.7] |
| 53-Mayenne | 413 | 309,168 | 137.6 | [124.3-150.9] | 204.5 | [181.9-227.2] | 66.4 | [53.3-79.5] |
| 54-Meurthe-et-Moselle | 1,476 | 735,062 | 202.7 | [192.4-213.1] | 280.2 | [263.3-297.2] | 120.3 | [108.8-131.7] |
| 55-Meuse | 369 | 192,867 | 189.4 | [170.0-208.8] | 265.5 | [233.3-297.7] | 108.3 | [87.8-128.9] |
| 56-Morbihan | 916 | 737,246 | 122.1 | [114.1-130.0] | 173.8 | [160.5-187.0] | 67.0 | [58.7-75.4] |
| 57-Moselle | 2,245 | 1,045,810 | 207.4 | [198.8-216.0] | 286.7 | [272.6-300.8] | 123.0 | [113.6-132.4] |
| 58-Nièvre | 331 | 216,568 | 146.7 | [130.5-162.9] | 210.7 | [183.6-237.9] | 78.6 | [61.9-95.3] |
| 59-Nord | 4,550 | 2,588,118 | 181.7 | [176.4-187.0] | 239.8 | [231.4-248.2] | 119.9 | [113.7-126.1] |
| 60-Oise | 1,507 | 809,140 | 188.4 | [178.9-198.0] | 263.7 | [248.0-279.5] | 108.3 | [98.0-118.6] |
| 61-Orne | 434 | 289,375 | 148.0 | [133.9-162.0] | 201.4 | [178.5-224.3] | 91.0 | [75.4-106.7] |
| 62-Pas-de-Calais | 2,981 | 1,464,038 | 205.8 | [198.5-213.2] | 281.4 | [269.4-293.4] | 125.4 | [117.1-133.7] |
| 63-Puy-de-Dôme | 885 | 639,947 | 135.8 | [126.8-144.7] | 196.1 | [181.1-211.1] | 71.6 | [62.3-80.8] |
| 64-Pyrénées-Atlantiques | 860 | 662,236 | 124.9 | [116.5-133.3] | 180.1 | [166.0-194.1] | 66.2 | [57.5-75.0] |
| 65-Hautes-Pyrénées | 320 | 229,096 | 134.4 | [119.5-149.3] | 197.4 | [172.1-222.7] | 67.3 | [52.3-82.4] |
| 66-Pyrénées-Orientales | 657 | 459,798 | 139.7 | [128.9-150.4] | 194.7 | [177.1-212.3] | 81.0 | [69.0-93.1] |
| 67-Bas-Rhin | 2,318 | 1,104,047 | 208.1 | [199.7-216.6] | 290.6 | [276.6-304.6] | 120.3 | [111.1-129.5] |
| 68-Haut-Rhin | 1,318 | 756,973 | 170.8 | [161.6-180.1] | 240.1 | [224.8-255.4] | 97.1 | [87.2-107.0] |
| 69-Rhône | 2,497 | 1,780,837 | 147.1 | [141.3-152.9] | 196.7 | [187.3-206.0] | 94.3 | [87.6-101.0] |
| 70-Haute-Saône | 439 | 240,160 | 178.1 | [161.4-194.8] | 255.1 | [227.1-283.1] | 96.1 | [78.7-113.5] |
| 71-Saône-et-Loire | 863 | 556,900 | 149.7 | [139.6-159.8] | 216.7 | [199.8-233.6] | 78.4 | [68.0-88.8] |
| 72-Sarthe | 930 | 569,029 | 165.1 | [154.5-175.8] | 241.3 | [223.4-259.2] | 84.0 | [73.2-94.8] |
| 73-Savoie | 609 | 425,412 | 141.4 | [130.2-152.6] | 194.6 | [176.2-213.0] | 84.7 | [72.4-97.1] |
| 74-Haute-Savoie | 971 | 767,473 | 125.2 | [117.3-133.1] | 178.3 | [165.2-191.5] | 68.6 | [60.3-77.0] |
| 75-Paris | 3,407 | 2,273,305 | 148.5 | [143.5-153.6] | 195.7 | [187.6-203.7] | 98.4 | [92.4-104.4] |
| 76-Seine-Maritime | 1,936 | 1,252,792 | 155.4 | [148.5-162.4] | 211.2 | [200.0-222.4] | 96.1 | [88.2-104.0] |
| 77-Seine-et-Marne | 2,007 | 1,361,739 | 152.1 | [145.4-158.8] | 210.9 | [199.8-221.9] | 89.5 | [82.2-96.9] |
| 78-Yvelines | 2,109 | 1,418,185 | 151.4 | [145.0-157.9] | 205.6 | [195.1-216.1] | 93.8 | [86.5-101.1] |
| 79-Deux-Sèvres | 434 | 374,151 | 114.1 | [103.3-124.9] | 169.2 | [150.8-187.6] | 55.4 | [44.8-66.0] |
| 80-Somme | 1,129 | 573,047 | 199.0 | [187.4-210.6] | 284.0 | [264.7-303.3] | 108.5 | [96.2-120.7] |
| 81-Tarn | 542 | 381,531 | 138.1 | [126.4-149.8] | 191.3 | [172.1-210.5] | 81.5 | [68.5-94.4] |
| 82-Tarn-et-Garonne | 359 | 250,334 | 141.6 | [126.9-156.3] | 198.6 | [174.2-222.9] | 81.0 | [65.1-96.8] |
| 83-Var | 1,441 | 1,019,910 | 135.7 | [128.6-142.8] | 188.4 | [176.8-199.9] | 79.7 | [71.8-87.5] |
| 84-Vaucluse | 825 | 551,898 | 146.3 | [136.3-156.3] | 203.4 | [187.0-219.8] | 85.5 | [74.5-96.6] |
| 85-Vendée | 839 | 657,326 | 126.3 | [117.7-134.9] | 180.9 | [166.5-195.4] | 68.1 | [59.2-77.0] |
| 86-Vienne | 619 | 430,677 | 144.5 | [133.1-155.9] | 207.9 | [188.8-226.9] | 77.0 | [65.1-89.0] |
| 87-Haute-Vienne | 539 | 377,482 | 139.1 | [127.3-150.9] | 194.2 | [174.8-213.7] | 80.4 | [67.6-93.2] |
| 88-Vosges | 695 | 376,918 | 179.5 | [166.1-192.9] | 254.0 | [231.8-276.3] | 100.1 | [85.9-114.2] |
| 89-Yonne | 627 | 342,060 | 180.1 | [166.0-194.3] | 267.7 | [243.6-291.7] | 86.9 | [72.9-101.0] |
| 90-Territoire de Belfort | 332 | 144,023 | 234.7 | [209.5-259.9] | 346.5 | [303.7-389.3] | 115.7 | [90.5-140.9] |
| 91-Essonne | 1,857 | 1,238,230 | 154.8 | [147.7-161.9] | 214.4 | [202.8-226.0] | 91.4 | [83.6-99.3] |
| 92-Hauts-de-Seine | 2,274 | 1,602,043 | 144.4 | [138.4-150.4] | 191.9 | [182.3-201.4] | 93.8 | [86.7-101.0] |
| 93-Seine-Saint-Denis | 1,880 | 1,545,357 | 131.0 | [124.9-137.0] | 179.1 | [169.2-189.0] | 79.7 | [72.9-86.5] |
| 94-Val-de-Marne | 2,138 | 1,348,290 | 162.6 | [155.6-169.5] | 218.2 | [207.0-229.4] | 103.3 | [95.3-111.4] |
| 95-Val-d'Oise | 1,741 | 1,191,214 | 153.7 | [146.4-161.0] | 211.9 | [200.0-223.9] | 91.7 | [83.6-99.9] |
| 971-Guadeloupe | 159 | 405,739 | 37.7 | [31.8-43.7] | 51.4 | [42.0-60.9] | 23.1 | [16.0-30.3] |
| 972-Martinique | 207 | 386,486 | 49.4 | [42.5-56.2] | 69.8 | [58.8-80.8] | 27.6 | [19.7-35.4] |
| 973-Guyane | 39 | 250,109 | 24.6 | [15.1-34.0] | 35.0 | [19.2-50.7] | 13.6 | [3.5-23.6] |
| 974-La Réunion | 177 | 840,974 | 22.9 | [19.3-26.4] | 26.7 | [21.3-32.0] | 18.8 | [14.2-23.5] |

^a^ Crude prevalence of MS. ^b^ Including 32 cases for which the region of residence was missing. Overall prevalence rates were standardised for age and gender, whereas gender-specific prevalence rates were standardised for age only.MS: multiple sclerosis.
